# The role of cognitive and brain reserve in memory decline and atrophy rate in mid and late-life: The SMART-MR study

**DOI:** 10.1016/j.cortex.2021.11.022

**Published:** 2022-01-31

**Authors:** Jet M.J. Vonk, Rashid Ghaznawi, Maarten H.T. Zwartbol, Yaakov Stern, Mirjam I. Geerlings

**Affiliations:** a Department of Epidemiology, Julius Center for Health Sciences and Primary Care, University Medical Center Utrecht and Utrecht University, Utrecht, the Netherlands; b Department of Neurology, Taub Institute for Research on Alzheimer's Disease and the Aging Brain, College of Physicians and Surgeons, Columbia University, New York, NY, USA; c Department of Radiology, University Medical Center Utrecht and Utrecht University, Utrecht, the Netherlands

**Keywords:** Cognitive reserve, Brain volume loss, Longitudinal, Neuroimaging, Cohort, Aging brain

## Abstract

**Objective:**

Investigate associations of cognitive and brain reserve with trajectories of memory decline in mid-life and late-life, and whether the relationship of memory decline with atrophy differs as a function of reserve.

**Methods:**

Participants were 989 Dutch middle-aged to older adults from the SMART-MR prospective cohort, followed up to 12 years with up to 3 measurements of memory and brain MRI. Education and Dutch National Adult Reading Test (DART) were used as proxies of cognitive reserve, and intracranial volume (ICV) and baseline brain parenchymal fraction (BPF) for brain reserve. Univariate growth curve models analyzed associations of reserve with memory decline, and multiple-group bivariate growth curve models tested the longitudinal brain–memory relationship as a function of reserve. Models were additionally stratified by mid-life and late-life.

**Results:**

Higher DART, education, and BPF were related to a slower rate of memory decline, particularly in late-life, but ICV was not. A positive covariance indicated that an individual who undergoes atrophy also undergoes memory decline—this relationship did not differ across cognitive or brain reserve, but was not present in mid-life. Memory declined slower than brain volume, yet rates were more similar in the low DART, education, and BPF groups.

**Discussion:**

Higher cognitive (DART, education) and brain reserve (BPF) work protectively in longitudinal memory change. ICV is an inappropriate proxy of brain reserve, failing to show any association with memory performance at baseline or over time. Deconstructing relationships of reserve capacities with longitudinal cognitive and brain outcomes may identify focus areas with potential for intervention.

## 1. Introduction

Cognitive aging embodies a large heterogeneity in levels of cognitive function and rates of cognitive change across individuals (Albert et al., 1995; Hayden et al., 2011; Mungas et al., 2010). This variability, which increases with advancing age (Ardila, 2007; Ylikoski et al., 1999), is not only present in individuals with clinical dementia (Wilkosz et al., 2010) or prodromal dementia (Panza et al., 2007) but also in individuals without dementia in mid-life (Singh-Manoux et al., 2011) and late-life (Zahodne et al., 2015). Among other factors, the heterogeneity has been attributed to differences in cognitive reserve and brain reserve (Groot et al., 2018; Singh-Manoux et al., 2011). In general, reserve capacity is thought to protect against clinical manifestation in the face of disease pathology. Cognitive reserve is the ability to maintain cognitive performance despite pathological disease burden through accumulated lifetime exposures (e.g., intelligence, education, social activities) (Stern, 2002; Stern et al., 2018). Brain reserve has been defined as ‘neurological capital,’ i.e., quantifiable brain resources (e.g., intracranial volume – ICV, synaptic count) that enhance or maintain cognitive function (Cabeza et al., 2018; Stern et al., 2018).

Positive associations of cognitive reserve with cognition have been shown extensively cross-sectionally (Cizginer et al., 2017; Rentz et al., 2017), but proxies of cognitive reserve often fail to show a positive relationship with longitudinal change (Lenehan et al., 2015; Soldan et al., 2017; Zahodne et al., 2011). For example, a recent meta-analysis by Seblova et al. (2020) showed that the association between education—the most commonly used proxy of cognitive reserve—and cognitive change over time was negligible. Studies on whether brain reserve benefits cognition cross-sectionally (Brickman et al., 2011; Cizginer et al., 2017) or longitudinally (Sumowski et al., 2014) have found mixed results. Notably, protective effects of either kind of reserve on the longitudinal relationship between brain change and memory change are relatively unexplored. This study sought to investigate 1) associations of cognitive reserve and brain reserve with trajectories of memory decline in mid-life and late-life, and 2) whether the relationship of brain volume loss over time (i.e., atrophy) with memory decline over time differs as a function of baseline cognitive reserve or brain reserve.

## 2. Method

### 2.1. Participants

Participants were drawn from the Second Manifestations of ARTerial diseases Magnetic Resonance (SMART-MR) study, a prospective cohort study among 1309 non-demented, independently living middle-aged to older adults with manifest arterial disease, which puts them at high risk for cognitive decline (Brickman et al., 2011). Individuals were assessed at up to three visits: at baseline (*n* = 1309^2^), after approximately 4 years (*n* = 754; retention rate visit 1 to 2 = 57.6%), and after 12 years (*n* = 329; retention rate visit 2 to 3 = 43.6%). Recruitment and procedures in SMART-MR have been described in detail elsewhere (Geerlings et al., 2010). The ethnicity of the participants is approximately 97% Caucasian (this includes people with Northern African (e.g., Moroccan) and Middle Eastern (e.g., Turkish) background, which is ~5.5% of the adult population in the Netherlands), 1% Black, and 1.5% South-East Asian. Participants in SMART-MR (*N* = 1309) were 20.3% women, partially reflecting differences in cardiovascular disease between men and women.

**Fig. 1 –:**
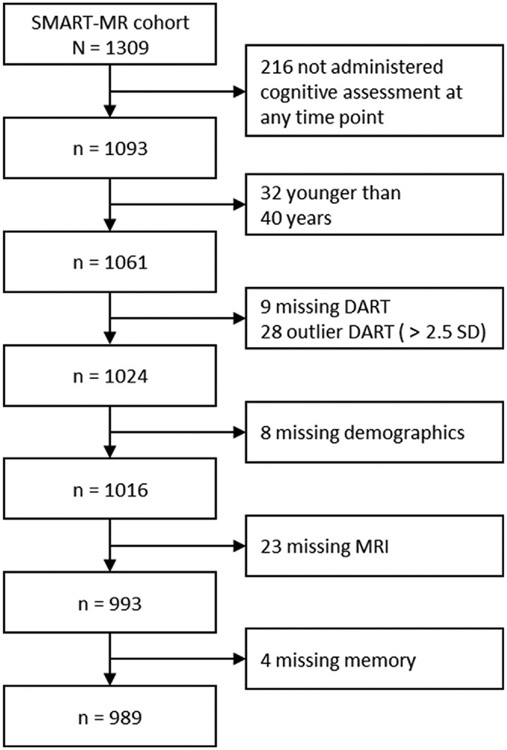
Flowchart of participant selection from the SMART-MR cohort.

We report how we determined our sample size, all data exclusions, all inclusion/exclusion criteria, whether inclusion/exclusion criteria were established prior to data analysis, all manipulations, and all measures in the study; Fig. 1 depicts a flowchart of participant selection. Table 1 displays an overview of the selected sample's characteristics at baseline (*n* = 989).

Written informed consent was obtained from all participants according to the principles of the Declaration of Helsinki and in accordance with the Medical Research Involving Human Subjects Act and the policies of the Medical Ethics Research Committee of the UMCU.

### 2.2. Cognitive measures

A domain score for memory was calculated as a composite measure of the total recall score and delayed recall score on the 15-word learning test [a modification of the Rey Auditory Verbal Learning test (Brand & Jolles, 1985)] and the delayed recall score of the Rey–Osterrieth Complex Figure test (Osterrieth, 1944). The three raw memory test scores at each wave were first converted into z-scores by subtracting the test's mean score of the study sample at baseline from each individual's score, and dividing by the standard deviation at baseline. Subsequently, the three standardized memory measures were averaged, and the resulting composite score was standardized.

### 2.3. Proxies of reserve

We used two proxies of cognitive reserve, education and the Dutch Adult Reading Test [DART (Schmand et al., 1998); Dutch version of the National Adult Reading Test, i.e., NART], and two proxies of brain reserve, ICV and baseline brain parenchymal fraction (BPF).

Education is the most commonly used proxy of cognitive reserve (Groot et al., 2018; Jones et al., 2011). Additionally, the DART, based on reading recognition of irregularly spelled words, is often used as a proxy of cognitive reserve (Soldan et al., 2017; Stern et al., 2005) as it is widely acknowledged to reflect a premorbid estimate of intellectual functioning (Schmand et al., 1998). This test is considered to be outside of the cognitive domains of language and memory, based on differential brain region activation patterns (Stern et al., 2003) as well as differential behavioral patterns (Joyce et al., 1996).

Total ICV is commonly used as a proxy of brain reserve in previous literature (Groot et al., 2018); ICV is considered to reflect maximal lifetime brain growth and remains stable during the course of neurodegeneration. BPF is the ratio of brain volume to ICV, and represents the volumetric status of the brain at the point in time of measurement. Thus, in terms of brain reserve, ICV could be considered as the fixed maximal brain reserve one could have had at some point in their life, while BPF at baseline could be considered as the available brain reserve at the start of the study.

### 2.4. MRI protocol and segmentation procedures

At all three waves, whole-brain brain images were obtained using a 1.5T Gyroscan ACS-NT Philips MRI scanner. The standardized scan protocol included transversal T1-weighted gradient-echo (38 contiguous slices; voxel size = .9 × .9 × 4.0 mm; field of view = 230 × 230 mm; matrix size = 180 × 256; flip angle = 80°; repetition time = 235 msec; echo time = 2 msec), T1-weighted inversion recovery (repetition time = 2900 msec; echo time = 22 msec; inversion time = 410 msec), T2-weighted (repetition time = 2200 msec; echo time = 11 msec), and FLAIR (repetition time = 6000 msec; echo time = 100 msec; inversion time = 2000 msec) MRI sequences.

Gray matter, white matter, sulcal and ventricular cerebrospinal fluid (CSF), and white matter hyperintensities (WMH) were segmented using the k-nearest neighbor classification technique (de Boer et al., 2010), performed on the T1-weighted gradient-echo, T2-weighted inversion recovery, and FLAIR images (Anbeek et al., 2005). WMH were additionally visually checked for correct segmentation using an image processing framework (MeVisLab 2.7.1., MeVis Medical Solutions AG, Bremen, Germany). If inspection showed that voxels were incorrectly segmented, they were added to the correct segmentation volumes using the image processing framework (Blom et al., 2019). Total brain volume was calculated as the sum of gray and white matter in the cerebrum, brainstem, and cerebellum, WMH, and cerebral infarcts volume. Total ICV was calculated at baseline as the sum of the total brain volume, and the volumes of sulcal and ventricular CSF.

BPF over time (i.e., total brain volume at each time point/ICV) was used as a measure of global brain atrophy across visits. This measure was standardized by subtracting the mean BPF at baseline from each individual's BPF at every visit, and dividing this value by the standard deviation of BPF at baseline. On average, men have a larger head size than women which affects ICV (Ruigrok et al., 2014); to adjust for this difference, ICV was standardized separately within men and within women. The mean ICV of men was subtracted from each man's ICV value, and divided by the standard deviation of men's ICV—and similarly for women.

### 2.5. Statistical analysis

Participants' characteristics were specified with descriptive statistics and differences between age groups (mid-life *vs* late-life) were tested with general linear models and chi-square tests in R version 3.5.1 (R Core Team, 2019) with the furniture package (Barrett & Brignone, 2017). Age groups were categorized based on whether one's age in the middle of their time period participating in the study fell above or below 60 years; as such, the majority of participants below this cut-off were followed for the most part in their mid-life, and those above the cut-off were mostly followed in their late-life. Level of education was assessed in seven levels, according to the Dutch educational system, which we categorized as less than high school education, at least some high school education, and a college/university degree.

To test associations of cognitive reserve and brain reserve with trajectories of memory decline, we estimated univariate growth curve models of memory performance over time with baseline DART, education, ICV, or baseline BPF (all as continuous, standardized measures) as a determinant of the intercept and slope of memory performance. Models were generated for the overall sample, as well as stratified by age group (mid-life or late-life). Time was parametrized as time in study (in years) with individually-varying time intervals. Covariates included age at baseline, sex/gender, and history of stroke. In all models, covariates were centered on the analysis’ sample to reflect main effects of average participants. The models additionally included a practice effect, often representing reduced anxiety on successive testing occasions. The practice effect was modeled through inclusion of a latent factor with memory performance fixed at the square root of the number of previous visits, i.e., with loadings fixed at 1 and 1.4 for the second and third time point, respectively (Vivot et al., 2016; Vonk et al., 2019, 2020). Lastly, all models were adjusted for potential selection bias due to missing data attributable to death by jointly modeling the survival process with the longitudinal process. Models included a latent hazard function to denote the conditional probability of death at a specific visit given survival and no drop-out at previous visits (i.e., a discrete-time survival analysis). This latent hazard function was regressed on the intercept and slope of the latent growth function to adjust the trajectory estimates for the potential effect of informative censoring.

Subsequently, we tested whether the relationship between atrophy and memory decline differed as a function of cognitive reserve (as proxied by DART or education) or brain reserve (as proxied by ICV or baseline BPF). First, we estimated a bivariate growth curve model, adjusted for age, sex/gender, and history of stroke, and assessed the covariance between standardized atrophy and standardized memory decline. We also compared the difference in these measures' rates of change with a Wald Test for equality of parameters. Next, we estimated the bivariate model as a multiple-group model for tertiles of DART, education, ICV, and baseline BPF, and compared whether the difference in the atrophy and memory decline measures' rates of change differed across low, mid, and high levels of these reserve variables with the Wald Test for equality of parameters. For DART, tertiles contained low DART = 35–75, mid DART = 76–89, and high DART = 90–100. For education, tertiles were categorized as low education = less than high school education, mid education = at least some high school education, and high education = a college/university degree. For ICV, tertiles were based on standardized scores due to sex/gender differences, and represented low ICV = −2.66 to −.46 SD, mid ICV = >−.46 to .40 SD, and high ICV = >.40 to 3.29 SD. For baseline BPF, tertiles included low baseline BPF = −3.65 to −.37 SD, mid baseline BPF = >−.37 to .51 SD, and high baseline BPF = >.51 thru 2.91 SD. The multiple-group models were not stratified by mid-life versus late-life due to low sample size when categorizing across both age groups and reserve groups.

All growth curve models were performed in Mplus version 6.12 (Muthén & Muthén, 1998–2011), and graphics were generated in R using ggplot2 (Wickham, 2016). We have included the syntax for all models in the Supplementary Material. No part of the study procedures or analyses was pre-registered prior to the research being conducted.

### 2.6. Data availability

For use of SMART-MR data, a request has to be made for UCC-SMART data (https://www.umcutrecht.nl/en/utrecht-cardiovascular-cohort). Please send an email to UCC data request (uccdatarequest@umcutrecht.nl). After registration, the administrator will send an invite which grants access to the data request module. The data are not publicly available due to privacy or ethical restrictions.

## 3. Results

### 3.1. Main effects of reserve on memory trajectories

#### 3.1.1. Cognitive reserve

**Fig. 2 –:**
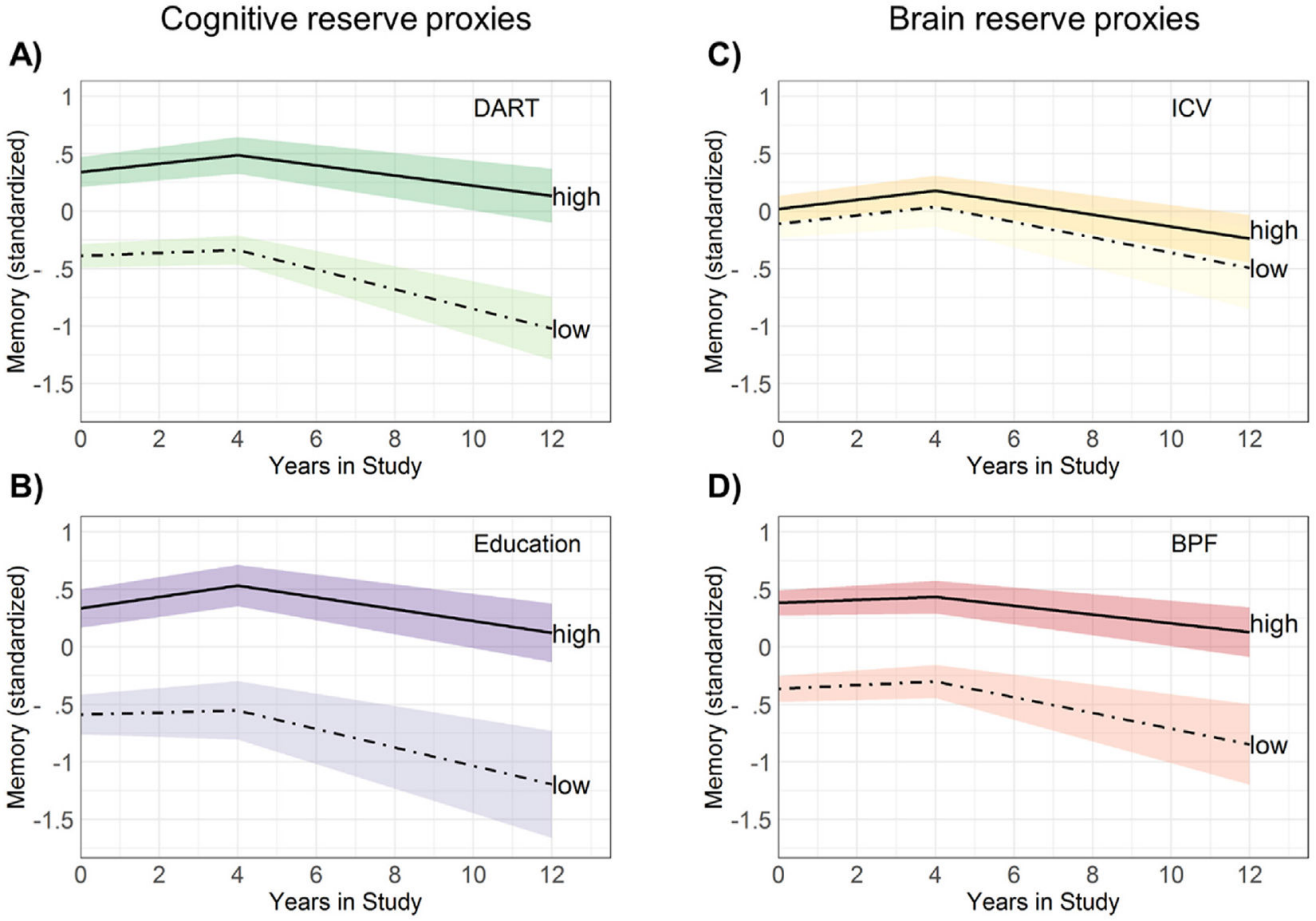
Trajectories of memory decline across high and low tertiles of A) Dutch Adult Reading Test (DART), B) education, C) intracranial volume (ICV), and D) brain parenchymal fraction (BPF).

Higher DART score was associated with better memory performance at baseline (Table 2; Fig. 2A). DART was also related to the slope of memory, such that a higher DART score was associated with slower memory decline compared to lower DART scores. When stratified by age group, a higher DART score related to better memory performance at baseline in both mid-life and late-life; however, only in late-life was a higher DART score related to slower memory decline. Education showed a similar pattern to DART score, where higher education was associated with better memory performance at baseline and slower memory decline overall and in the late-life stratum, but no effect of education on the slope of decline in mid-life (Table 2; Fig. 2B). The correlation between DART and education was *r* = .502, *p* < .001.

#### 3.1.2. Brain reserve

ICV was not associated with memory performance at baseline, nor did it affect the slope of memory performance over time (Table 2; Fig. 2C). The same pattern was observed when stratified across mid-life and late-life. BPF at baseline as a measure of brain reserve was associated with better memory at baseline in the overall sample, but not when stratified across age groups (Table 2; Fig. 2D). Higher BPF at baseline was associated with slower memory decline compared to lower BPF at baseline. When stratified by age group, higher BPF at baseline was related to slower memory decline in late-life but not in mid-life.

### 3.2. Rate of memory versus atrophy by reserve capacity

Memory declined at a rate of −.085 ([−.104, −.066], *p* < .001) standardized scores per year, while brain volume declined at a rate of −.137 ([−.146, −.129], *p* < .001) standardized measurements per year. As such, memory declined slower than brain volume (Δ*B* = .052 [.033, .071], *p* < .001). There was a positive covariance between decline in memory and atrophy (cov = .001,^3^ *p* = .018), indicating that an individual who undergoes atrophy also undergoes memory decline. When stratified, memory (mid-life: *B* = −.057 [−.096, −.018], *p* = .004; late-life: *B* = −.115 [−.144, −.086], *p* < .001) and brain volume (mid-life: *B* = −.117 [−.136, −.099], *p* < .001; late-life: *B* = −.156 [−.168, −.143], *p* < .001) declined across both age strata. A slower decline of memory than brain volume was also observed across both mid-life (Δ*B* = .060 [.013, .108], *p* = .013) and late-life (Δ*B* = .040 [.010, .070], *p* = .009). While the rates of decline covaried in late-life (cov = .001, *p* = .028), they did not in mid-life (cov < .001, *p* = .882).

#### 3.2.1. Cognitive reserve

**Fig. 3 –:**
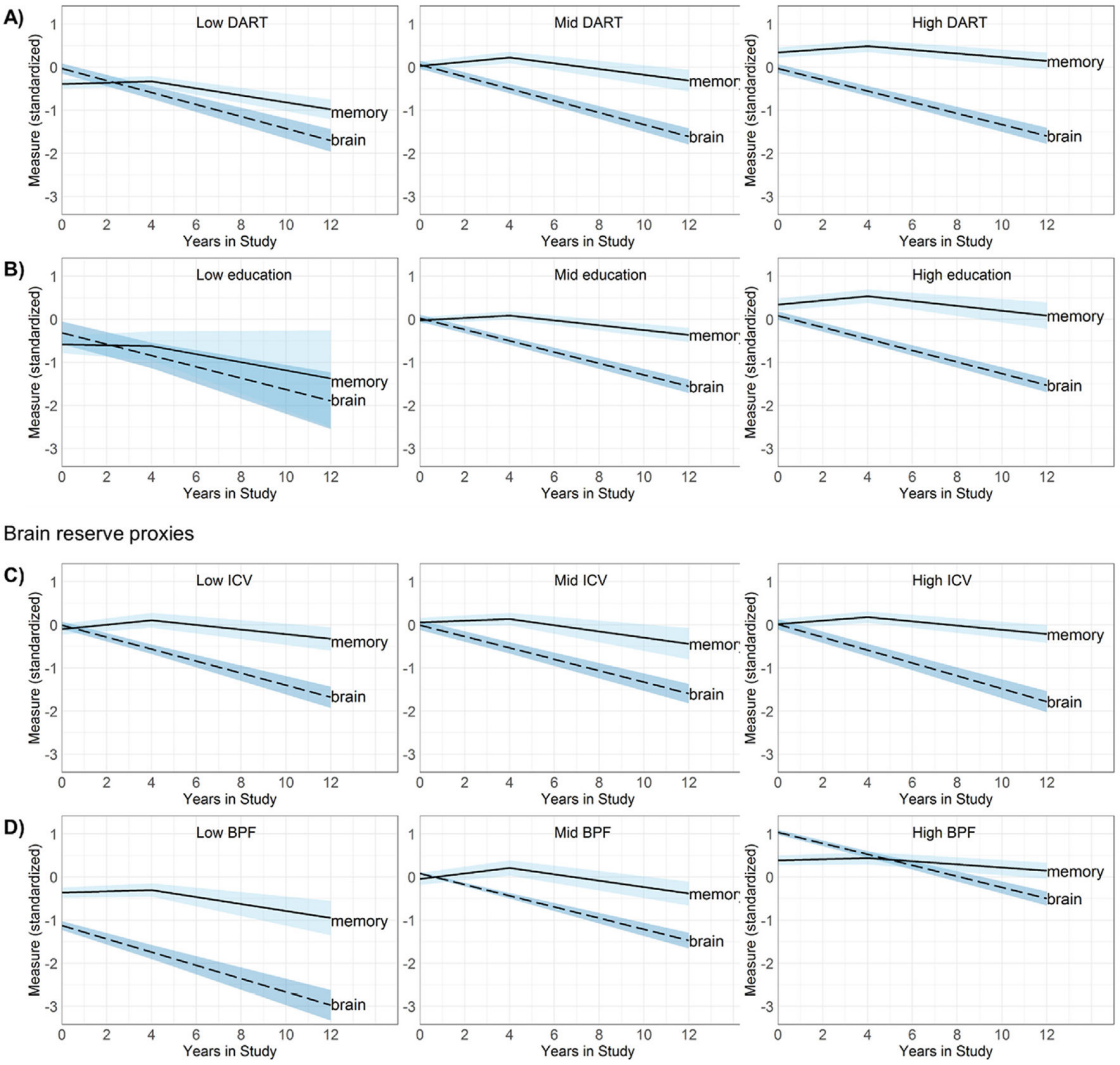
Rate of memory decline versus rate of brain volume loss per tertiles of A) Dutch Adult Reading Test (DART), B) education, C) intracranial volume (ICV), and D) brain parenchymal fraction (BPF).

The pattern that memory declined slower than brain volume was present in the mid DART (Δ*B* = .043 [.008, .078], *p* = .016) and high DART (Δ*B* = .067 [.035, .098], *p* < .001) groups, but the decline was at more similar rates in the low DART group (Δ*B* = .033 [−.007, .074], *p* = .108; Fig. 3A). Similarly, the pattern of slower memory decline than atrophy was present in the mid education (Δ*B* = .054 [.029, .079], *p* < .001) and high education groups (Δ*B* = .053 [.011, .094], *p* = .013), while rates were more similar in the low education group (Δ*B* = .016 [−.137, .168], *p* = .840; Fig. 3B).

Formally testing differences among the DART groups showed that the relationship of brain volume loss over time with memory decline did not differ as a function of DART group (low *vs* mid *p* = .726; low *vs* high *p* = .204; mid *vs* high *p* = .319). The covariance between memory decline and brain volume loss also did not differ between DART groups (low *vs* mid *p* = .827; low *vs* high *p* = .439; mid *vs* high *p* = .511). Brain volume declined at similar rates across DART groups (low *vs* mid *p* = .971; low *vs* high *p* = .439; mid *vs* high *p* = .380; Table 3). Memory also declined at similar rates across DART groups (low *vs* mid *p* = .698; low *vs* high *p* = .084; mid *vs* high *p* = .156; Table 3).

There were also no differences in the relationship between brain and memory decline among the three education groups (low *vs* mid *p* = .628; low *vs* high *p* = .648; mid *vs* high *p* = .952), nor in their covariance (low *vs* mid *p* = .533; low *vs* high *p* = .547; mid *vs* high *p* = .935). Brain volume declined at similar rates between education groups (low *vs* mid *p* = .991; low *vs* high *p* = .916; mid *vs* high *p* = .740; Table 3). Memory also declined at similar rates between education groups (low *vs* mid *p* = .629; low *vs* high *p* = .675; mid *vs* high *p* = .864; Table 3).

#### 3.2.2. Brain reserve

The pattern that memory declined slower than brain volume was present in the low ICV (Δ*B* = .054 [.017, .091], *p* = .004) and high ICV groups (Δ*B* = .078 [.044, .112], *p* < .001), but brain and memory declined at more similar rates in the mid ICV group (Δ*B* = .038 [−.014, .089], *p* = .149; Fig. 3C). Within baseline BPF groups, memory declined slower than brain volume in the high baseline BPF group (Δ*B* = .079 [.048, .110], *p* < .001), but the decline was at more similar rates in the low baseline BPF (Δ*B* = .049 [−.010, .109], *p* = .104) and mid baseline BPF groups (Δ*B* = .022 [−.009, .052], *p* = .164; Fig. 3D).

The relationship between the slope of brain volume and the slope of memory did not differ between the ICV groups (low *vs* mid *p* = .616; low *vs* high *p* = .351; mid *vs* high *p* = .203), nor did the covariance between the two slopes between the groups (low *vs* mid *p* = .692; low *vs* high *p* = .949; mid *vs* high *p* = .714). Brain volume declined at similar rates between ICV groups (low *vs* mid *p* = .621; low *vs* high *p* = .431; mid *vs* high *p* = .148; Table 3). Memory also declined at similar rates between ICV groups (low *vs* mid *p* = .762; low *vs* high *p* = .580; mid *vs* high *p* = .447; Table 3).

For baseline BPF, no difference was observed in the relationship between brain volume loss and memory decline between the low versus mid groups (*p* = .417) and low versus high groups (*p* = .389), but the difference in rate of decline between brain and memory was larger in the high than the mid baseline BPF group (*p* = .010) with slower memory than brain volume decline. There was no difference in the slopes' covariance between the groups (low *vs* mid *p* = .412; low *vs* high *p* = .238; mid *vs* high *p* = .589). There were no differences between baseline BPF groups in rate of atrophy (low *vs* mid *p* = .115; low *vs* high *p* = .074; mid *vs* high *p* = .842). Note that memory declined slower in the high than mid baseline BPF group (*p* = .007), while no differences were observed between the low versus mid (*p* = .921) and low versus high groups (*p* = .085; Table 3).

## 4. Discussion

This study investigated relationships among cognitive reserve, brain reserve, memory decline, and atrophy in cognitively normal adults in mid-life and late-life. Results showed that the cognitive reserve proxies of DART and education, and the brain reserve proxy of baseline BPF were related to the rate of memory decline—particularly in late-life—but the popular brain reserve proxy of ICV was not. We found that memory decline and atrophy simultaneously unfold over time (i.e., they covaried), but that the rate of memory decline was slower than the rate of atrophy, yet at more similar rates in the low cognitive reserve (DART and education) and brain reserve (baseline BPF) groups. These findings contribute to a better understanding of the heterogeneity within cognitive change in relation to brain change.

Our finding that higher cognitive reserve, proxied by either DART or education, was related to a slower rate of memory decline counters previous studies that did not find a protective effect of cognitive reserve on longitudinal change in memory performance (Seblova et al., 2020; Soldan et al., 2017; Zahodne et al., 2011). The majority of these studies used education as a proxy of cognitive reserve, as shown in the meta-analysis by Seblova et al. (2020), but the appropriateness of this measure as a proxy of reserve has been debated (Jones et al., 2011). Instead, other measures such as literacy and premorbid intellectual functioning have been proposed as better proxies (Manly et al., 2005; Stern, 2006). Our sample shows effects for both DART—a measure of premorbid intellectual functioning—and education as a proxy for cognitive reserve. Any proxy of cognitive reserve, however, should be employed with caution and not be interpreted as a direct measure of cognitive reserve (Stern et al., 2018). Future research should investigate the relationship of different measurements of cognitive reserve with longitudinal cognitive decline, including dynamic measurements of cognitive reserve as opposed to static proxies (Bettcher et al., 2019). Dynamic measurements of cognitive reserve are composed by modeling reserve capacity as residual cognition not explained by demographic and brain variables, i.e., one's cognitive performance beyond what is expected based on demographic and brain variables (Reed et al., 2010).

The rate of brain volume loss was parallel across cognitive reserve groups, but slower decline of memory with higher cognitive reserve compared to lower cognitive reserve resulted in a larger gap in rates of brain and memory change in the high cognitive reserve group compared to the low cognitive reserve group. This finding is in line with the expectations posed by Stern et al. (2018), who proposed that individuals with high cognitive reserve have a greater ability to adapt to brain changes than those with low cognitive reserve, which in turn affects their cognition less.

Brain reserve as proxied by ICV was not associated with memory decline over time, nor baseline memory performance—the opposite results of what we found for the two proxies of cognitive reserve. The results may suggest that ICV is not an appropriate proxy of brain reserve: ICV failed to show any association with memory performance in any of the analyses conducted in this study. Specifically, ICV was not related to baseline memory performance for the overall sample nor either age group, while conceptually, a proxy of brain reserve should be related to clinical or cognitive outcomes (Stern, 2002). We should note, however, that cognitive ability includes more domains besides memory, and other domains could possibly be more strongly related to ICV. The differential relations of ICV across various cognitive domains should be investigated in more detail in future research.

ICV is an approximation of the fixed maximal brain reserve one had when their brain volume was at its maximum, but does not reflect one's available brain reserve at the time of cognitive assessment. Yet, the idea of reserve capacity is that it is not fixed or immutable, but can grow through lifetimes experiences (Stern et al., 2018). Similarly, years of education is an approximation of the fixed maximal cognitive reserve one had after obtaining their maximum educational attainment. Therefore, relatively static proxies like education for cognitive reserve and ICV for brain reserve may not accurately capture one's reserve capacity at the time of cognitive assessment. While education mimics the patterns of DART in our sample due to their high correlation, larger variation (i.e., more uncertainty towards the effect on memory) is observed around estimates for the low education group—possibly due to the unequal spread of number of participants across education groups. Collectively, adaptable proxies such as DART and baseline BPF, other dynamic proxies of reserve, or composite/factor scores of reserve that include multiple life-time experiences are preferred measures of reserve capacity over static and/or single proxies like education and ICV.

Brain reserve as proxied by baseline BPF showed similar results to the two cognitive reserve proxies. Several researchers have argued that the distinction between cognitive reserve and brain reserve is somewhat artificial and that these terms represent the same underlying construct, as both are brain-based (Cabeza et al., 2018; Jones et al., 2010). Our finding of a similar pattern for baseline BPF as for DART and education in relation to memory decline over time does not allow us to draw any conclusions regarding whether these concepts may or may not represent different constructs of reserve; more research is needed that conceptually separates these types in testing hypotheses related to reserve mechanisms.

Typically, studies on cognitive aging and the influence of cognitive reserve and brain reserve are aimed at investigating processes in late-life, but the processes of (subtle) cognitive and brain decline may already start in mid-life (Debette et al., 2009; Singh-Manoux et al., 2011). A strength of our study is that we investigated both mid-life and early late-life separately. While most patterns of memory and brain decline in relation to cognitive reserve and brain reserve were observed in both groups, the two groups differed in that DART, education, and baseline BPF were only related to the rate of memory decline in late-life. This result does not seem to be linked to the amount of memory decline across groups, as both groups displayed clear patterns of a decrease in memory performance over time. We suspect that the protective effect of cognitive reserve may be more noticeable in late-life, as cognitive reserve represents the ability to maintain cognitive function in the face of disease, and age is the largest risk factor for dementia (Guerreiro & Bras, 2015). As such, it is worthwhile to carefully consider relationships across different stages of life in research on protective factors in aging and dementia (Brayne, 2007).

We also recognize several limitations of our study. We focused on the relationship between memory performance and global brain volume. Future studies may investigate more cognitive domains as well as different brain measurements that could provide more insight into the associations of cognitive reserve and brain reserve with longitudinal outcomes—brain measures that are more directly associated with memory (e.g., hippocampal volume or regional cortical thickness) may be more likely to test a (stronger) moderation effect of cognitive reserve. Directions to explore may include inter-individual differences in the dynamic relationships between various measurements of neurodegeneration and cognitive functioning. Another limitation pertains to the underrepresentation of women and racial or ethnic groups in the SMART-MR cohort; the asymmetry in representation of sex/gender and race/ethnicity may limit the generalizability of results in this cohort to the general population. Generalizability may also be limited by the clinic-based nature of the SMART-MR cohort that is focused on individuals with manifest arterial disease. Additionally, the cohort experienced loss to follow-up, but reasons for attrition other than death (e.g., comorbidity) were unknown—while we adjusted for potential selection bias due to death, we were unable to assess whether there may have been informative censoring due to other reasons than death.

This study on longitudinal changes in cognition and brain volume responds to the need to better understand inter-individual as well as intra-individual differences in cognitive aging (Stern et al., 2018). The knowledge gained by this line of research has important implications; deconstructing the relationships of cognitive reserve and brain reserve with longitudinal cognitive and brain outcomes could identify focus areas that have potential for intervention, particularly among individuals at greater risk for cognitive decline. For example, public policies to improve individuals' cognitive reserve capacities may allow some individuals to maintain normal cognitive performance longer despite the presence of neuro-degenerative disease pathology. For such early behavioral intervention, future research on cognitive reserve and brain reserve will be particularly valuable in individuals without dementia who may be at high risk or in a preclinical phase, including those in their mid-life years.

## Supplementary Material

Supplementary

## Figures and Tables

**Table 1 –:** Participant characteristics at baseline.

	Whole sample	Mid-life	Late-life	*P* value
n	989	436	553	
Age at baseline	58.7 (9.1, 40–79)	50.5 (4.8, 40–59)	65.2 (5.9, 54–79)	<.001
Sex/gender (women)	206 (20.8%)	90 (20.6%)	116 (21%)	.960
Education				
Less than high school	116 (11.7%)	40 (9.2%)	76 (13.7%)	.066
High school	640 (64.7%)	295 (67.7%)	345 (62.4%)	
College/university	233 (23.6%)	101 (23.2%)	132 (23.9%)	
History of stroke	107 (10.8%)	48 (11%)	59 (10.7%)	.946
DART score at baseline	79.5 (15.0, 35–100)	79.8 (13.9, 37–100)	79.4 (15.8, 35–100)	.675
APOE ε4 allele carrier	301 (30.4%)	143 (32.8%)	158 (28.6%)	.153
ICV at baseline	1461.2 (129.3, 1092.2–1887.1)	1460.5 (130.1, 1135.6–1887.1)	1461.7 (128.8, 1092.2–1803.7)	.890
BPF at baseline	.79 (.03, .69–.87)	.80 (.02, .72–.87)	.78 (.03, .69–.84)	<.001
Total recall 15-item word-list	37.2 (9.7, 13–64)	40.1 (9.4, 18–64)	34.9 (9.3, 13–63)	<.001
Delayed recall 15-item word-list	7.2 (2.9, 1–15)	7.9 (2.9, 1–15)	6.5 (2.7, 1–15)	<.001
Rey delayed recall	19.7 (7.0, 0–36)	21.5 (6.6, 4–36)	18.3 (7.0, 0–36)	<.001
Number of visits	1.8 (.8, 1–3)	1.7 (.7, 1–3)	1.8 (.8, 1–3)	.236
Time in study (in years)	5.4 (4.8, 0–13.6)	5.2 (4.8, 0–13.6)	5.6 (4.8, 0–13.2)	.138

*Note*. Categorical: *n* (%), continuous: mean (standard deviation; range); DART = Dutch Adult Reading test; ICV = intracranial volume; BPF = brain parenchymal fraction.

**Table 2 –:** Main effects on the intercept and slope of memory performance of Dutch Adult Reading Test (DART) and education as proxies of cognitive reserve, and intracranial volume (ICV) and brain parenchymal fraction (BPF) at baseline as proxies of brain reserve.

	Intercept	Slope
*Overall sample*		
DART	.350 [.295, .406]*	.008 [.001, .015]*
Education	.242 [.179, .304]*	.008 [.001, .015]*
ICV	.033 [−.028 .095]	.000 [−.007, .007]
BPF	.076 [.000, .152]*	.017 [.009, .026]*
*Mid-life*		
DART	.355 [.269, .442]*	.000 [−.007, .008]
Education	.247 [.155, .339]*	.001 [−.008, .011]
ICV	.005 [−.079, .090]	.001 [−.009, .012]
BPF	.094 [−.029, .217]	−.015 [−.034, .003]
*Late-life*		
DART	.351 [.278, .424]*	.011 [.002, .019]*
Education	.247 [.161, .333]*	.011 [.001, .020]*
ICV	.053 [−.035, .141]	.000 [−.010, .009]
BPF	.091 [−.011, .194]	.021 [.007, .034]*

*Note*. DART = Dutch Adult Reading test; ICV = intracranial volume; BPF = brain parenchymal fraction; estimates represent: standardized parameter estimate [95% confidence interval]; *p < .05.

**Table 3 –:** Rates of memory decline and brain volume loss for the overall sample per reserve group of Dutch Adult Reading Test (DART) and education as proxies of cognitive reserve, and intracranial volume (ICV) and brain parenchymal fraction (BPF) at baseline as proxies of brain reserve.

Reserve measure	Group	Slope of	Estimate
DART	Low (*n* = 330)	Memory	−.106 [−.143, −.068]*
		Brain	−.139 [−.157, −.121]*
	Mid (*n* = 341)	Memory	−.096 [−.129, −.062]*
		Brain	−.139 [−.152, −.125]*
	High (*n* = 318)	Memory	−.063 [−.093, −.033]*
		Brain	−.130 [−.144, −.116]*
Education	Low (*n* = 116)	Memory	−.116 [−.267, .036]
		Brain	−.131 [−.188, −.075]*
	Mid (*n* = 640)	Memory	−.078 [−.102, −.053]*
		Brain	−.132 [−.143, −.120]*
	High (*n* = 233)	Memory	−.082 [−.124, −.040]*
		Brain	−.134 [−.146, −.123]*
ICV	Low (*n* = 330)	Memory	−.085 [−.122, −.048]*
		Brain	−.139 [−.159, −.119]*
	Mid (*n* = 330)	Memory	−.095 [−.146, −.044]*
		Brain	−.133 [−.149, −.116]*
	High (*n* = 329)	Memory	−.071 [−.103, −.040]*
		Brain	−.149 [−.165, −.134]*
BPF	Low (*n* = 328)	Memory	−.161 [−.105, −.049]*
		Brain	−.180 [−.154, −.128]*
	Mid (*n* = 332)	Memory	−.108 [−.139, −.078]*
		Brain	−.130 [−.145, −.115]*
	High (*n* = 329)	Memory	−.049 [−.079, −.019]*
		Brain	−.128 [−.140, −.116]*

Note. Estimates represent: standardized parameter estimate [95% confidence interval]; *p < .05.

## Footnotes

2 Because neuropsychological assessment was first started two years after the cohort's start, 480 individuals did not receive cognitive testing at baseline—264 of those did receive cognitive testing at follow-up.

3 A covariance is an unstandardized value from which it is not possible to determine the strength of a relationship; because the model is run on standardized measures with a mean of 0, the covariance value is also small. Due to the model's complexity, we were unable to normalize the covariance to a Pearson's correlation coefficient.
