# Supplementary material for: The role of cognitive and brain reserve in memory decline and atrophy rate in mid and late-life: The SMART-MR study
Source: Cortex. Author manuscript; Available in PMC 2024 Apr 15. (PMC11018269; doi:10.1016/j.cortex.2021.11.022)
Supplement: Supplementary [file NIHMS1979219-supplement-Supplementary.docx]

Supplementary Material

# Model 1a. Univariate model of DART on memory

VARIABLE: NAMES = id sex stroke age agegr T1 T2 T3 edu ZageT1 Znlv Zeducat ZICV nlv_t ICV_t zBPF1 zBPF2 zBPF3 BPF_t DDV2 DDV3 zmem1 zmem2 zmem3 age60 r2 r3 r3t;

usevariables are id zmem1 zmem2 zmem3 T1-T3 age sex DDV2 DDV3 Znlv stroke;

!USEOBSERVATIONS ARE age60 EQ 0;

IDVARIABLE = id;

MISSING ARE ALL (-999) ;

TSCORES= T1 T2 T3;

Centering = GRANDMEAN (age sex stroke);

ANALYSIS: TYPE=RANDOM;

ESTIMATOR = MLF;

MODEL:

i1 s1 | zmem1 zmem2 zmem3 AT T1-T3;

[i1*];

[s1*] (s1);

i1*;

s1*;

retest BY zmem2@1 zmem3@1.4;

[retest*];

retest@0;

retest with i1@0 s1@0;

i1 s1 ON age sex stroke;

i1 s1 ON Znlv;

F BY DDV2@1 DDV3@1;

F@0;

F ON I1;

F ON S1;

output: CINTERVAL;

# Model 1b. Univariate model of education on memory

VARIABLE: NAMES = id sex stroke age agegr T1 T2 T3 edu ZageT1 Znlv Zeducat ZICV nlv_t ICV_t zBPF1 zBPF2 zBPF3 BPF_t DDV2 DDV3 zmem1 zmem2 zmem3 age60 r2 r3 r3t;

usevariables are id zmem1 zmem2 zmem3 T1-T3 age sex DDV2 DDV3 Zeducat stroke;

!USEOBSERVATIONS ARE age60 EQ 0;

IDVARIABLE = id;

MISSING ARE ALL (-999) ;

TSCORES= T1 T2 T3;

Centering = GRANDMEAN (age sex stroke);

ANALYSIS: TYPE=RANDOM;

ESTIMATOR = MLF;

MODEL:

i1 s1 | zmem1 zmem2 zmem3 AT T1-T3;

[i1*];

[s1*] (s1);

i1*;

s1*;

retest BY zmem2@1 zmem3@1.4;

[retest*];

retest@0;

retest with i1@0 s1@0;

i1 s1 ON age sex stroke;

i1 s1 ON Zeducat;

F BY DDV2@1 DDV3@1;

F@0;

F ON I1;

F ON S1;

output: CINTERVAL;

# Model 2a. Univariate model of ICV on memory

VARIABLE: NAMES = id sex stroke age agegr T1 T2 T3 edu ZageT1 Znlv Zeducat ZICV nlv_t ICV_t zBPF1 zBPF2 zBPF3 BPF_t DDV2 DDV3 zmem1 zmem2 zmem3 age60 r2 r3 r3t;

usevariables are id zmem1 zmem2 zmem3 T1-T3 age sex DDV2 DDV3 ZICV stroke;

!USEOBSERVATIONS ARE age60 EQ 0;

IDVARIABLE = id;

MISSING ARE ALL (-999) ;

TSCORES= T1 T2 T3;

Centering = GRANDMEAN (age sex stroke);

ANALYSIS: TYPE=RANDOM;

ESTIMATOR = MLF;

MODEL:

i1 s1 | zmem1 zmem2 zmem3 AT T1-T3;

[i1*];

[s1*] (s1);

i1*;

s1*;

retest BY zmem2@1 zmem3@1.4;

[retest*];

retest@0;

retest with i1@0 s1@0;

i1 s1 ON age sex stroke;

i1 s1 ON ZICV;

F BY DDV2@1 DDV3@1;

F@0;

F ON I1;

F ON S1;

output: CINTERVAL;

# Model 2b. Univariate model of BPF on memory

VARIABLE: NAMES = id sex stroke age agegr T1 T2 T3 edu ZageT1 Znlv Zeducat ZICV nlv_t ICV_t zBPF1 zBPF2 zBPF3 BPF_t DDV2 DDV3 zmem1 zmem2 zmem3 age60 r2 r3 r3t;

usevariables are id zmem1 zmem2 zmem3 T1-T3 age sex DDV2 DDV3 zBPF1 stroke;

!USEOBSERVATIONS ARE age60 EQ 0;

IDVARIABLE = id;

MISSING ARE ALL (-999) ;

TSCORES= T1 T2 T3;

Centering = GRANDMEAN (age sex stroke);

ANALYSIS:TYPE=RANDOM;

ESTIMATOR = MLF;

MODEL:

i1 s1 | zmem1 zmem2 zmem3 AT T1-T3;

[i1*];

[s1*] (s1);

i1*;

s1*;

retest BY zmem2@1 zmem3@1.4;

[retest*];

retest@0;

retest with i1@0 s1@0;

i1 s1 ON age sex stroke;

i1 s1 ON zBPF1;

F BY DDV2@1 DDV3@1;

F@0;

F ON I1;

F ON S1;

output: CINTERVAL;

# Model 3a. Bivariate model of the overall sample

VARIABLE: NAMES = id sex stroke age agegr T1 T2 T3 edu ZageT1 Znlv Zeducat ZICV nlv_t ICV_t zBPF1 zBPF2 zBPF3 BPF_t DDV2 DDV3 zmem1 zmem2 zmem3 age60 r2 r3 r3t;

usevariables are id zmem1 zmem2 zmem3 T1-T3 zBPF1 zBPF2 zBPF3 age sex DDV2 DDV3 stroke;

!USEOBSERVATIONS ARE age60 EQ 0;

!GROUPING = nlv_t (0=low 1=mid 2=high);

IDVARIABLE = id;

MISSING ARE ALL (-999) ;

TSCORES= T1 T2 T3;

Centering = GRANDMEAN (age sex stroke);

ANALYSIS:TYPE=RANDOM;

ESTIMATOR = MLF;

MODEL:

i1 s1 | zmem1 zmem2 zmem3 AT T1-T3;

[i1*] (i1);

[s1*] (s1);

i1*;

s1*;

retest BY zmem2@1 zmem3@1.4;

[retest*] (retest);

retest@0;

retest with i1@0 s1@0;

i2 s2 | zBPF1 zBPF2 zBPF3 AT T1-T3;

[i2*] (i2);

[s2*] (s2);

i2*;

s2*;

i1 s1 i2 s2 ON age sex stroke;

i1 with i2 s1 s2;

s1 with i2 s2;

i2 with s2;

F BY DDV2@1 DDV3@1;

F@0;

F ON I1 i2;

F ON S1 s2;

MODEL CONSTRAINT:

new(sdif);

sdif = s1-s2;

new(T0l T4l T12l T0h T4h T12h);

T0L = (i1)+(s1)*(0);

T4L = (i1)+(s1)*(4)+retest*4;

T12L = (i1)+(s1)*(12)+retest*12;

T0H = (i2)+(s2)*(0);

T4H = (i2)+(s2)*(4);

T12H = (i2)+(s2)*(12);

output: CINTERVAL;

# Model 4a. Bivariate multiple group model

VARIABLE: NAMES = id sex stroke age agegr T1 T2 T3 edu ZageT1 Znlv Zeducat ZICV nlv_t ICV_t zBPF1 zBPF2 zBPF3 BPF_t DDV2 DDV3 zmem1 zmem2 zmem3 age60 r2 r3 r3t;

usevariables are id zmem1 zmem2 zmem3 T1-T3 zBPF1 zBPF2 zBPF3 age sex DDV2 DDV3 stroke;

!USEOBSERVATIONS ARE age60 EQ 0;

GROUPING = nlv_t (0=low 1=mid 2=high);

IDVARIABLE = id;

MISSING ARE ALL (-999) ;

TSCORES= T1 T2 T3;

Centering = GRANDMEAN (age sex stroke);

ANALYSIS:TYPE=RANDOM;

ESTIMATOR = MLF;

MODEL:

i1 s1 | zmem1 zmem2 zmem3 AT T1-T3;

[i1*] ;

[s1*] ;

i1*;

s1*;

retest BY zmem2@1 zmem3@1.4;

[retest*];

retest@0;

retest with i1@0 s1@0;

i2 s2 | zBPF1 zBPF2 zBPF3 AT T1-T3;

[i2*] ;

[s2*] ;

i2*;

s2*;

i1 s1 i2 s2 ON age sex stroke;

i1 with i2 s1 s2;

s1 with i2 s2;

i2 with s2;

F BY DDV2@1 DDV3@1;

F@0;

F ON I1 i2;

F ON S1 s2;

MODEL LOW:

[i1*] (i1low);

[s1*] (s1low);

i1*;

s1*;

retest BY zmem2@1 zmem3@1.4;

[retest*] (rlow);

retest@0;

retest with i1@0 s1@0;

[i2*] (i2low);

[s2*] (s2low);

i2*;

s2*;

i1 s1 i2 s2 ON age sex stroke;

s1 with s2 (scovLCR);

i1 with i2 s1 s2;

i2 with s2 s1;

F BY DDV2@1 DDV3@1;

F@0;

F ON I1 i2;

F ON S1 s2;

MODEL MID:

[i1*] (i1mid);

[s1*] (s1mid);

i1*;

s1*;

retest BY zmem2@1 zmem3@1.4;

[retest*] (rmid);

retest@0;

retest with i1@0 s1@0;

[i2*] (i2mid);

[s2*] (s2mid);

i2*;

s2*;

i1 s1 i2 s2 ON age sex stroke;

s1 with s2 (scovMCR);

i1 with i2 s1 s2;

i2 with s2 s1;

F BY DDV2@1 DDV3@1;

F@0;

F ON I1 i2;

F ON S1 s2;

MODEL HIGH:

[i1*] (i1hi);

[s1*] (s1hi);

i1*;

s1*;

retest BY zmem2@1 zmem3@1.4;

[retest*] (rhi);

retest@0;

retest with i1@0 s1@0;

[i2*] (i2hi);

[s2*] (s2hi);

i2*;

s2*;

i1 s1 i2 s2 ON age sex stroke;

s1 with s2 (scovHCR);

i1 with i2 s1 s2;

i2 with s2 s1;

F BY DDV2@1 DDV3@1;

F@0;

F ON I1 i2;

F ON S1 s2;

MODEL CONSTRAINT:

new(sCR_LM sCR_LH sCR_MH

difslow difsmid difshi

brainlm brainlh brainmh memlm memlh

memmh midlow hilow midhi);

sCR_LM = scovMCR-scovLCR;

sCR_LH = scovHCR-scovLCR;

sCR_MH = scovHCR-scovMCR;

difslow = s1low-s2low;

difsmid = s1mid-s2mid;

difshi = s1hi-s2hi;

brainlm = s2low-s2mid;

brainlh = s2low-s2hi;

brainmh = s2mid-s2hi;

memlm = s1low-s1mid;

memlh = s1low-s1hi;

memmh = s1mid-s1hi;

midlow = difslow-difsmid;

hilow = difslow-difshi;

midhi = difsmid-difshi;

new(T0L1 T4L1 T12L1

T0L2 T4L2 T12L2

T0M1 T4M1 T12M1

T0M2 T4M2 T12M2

T0H1 T4H1 T12H1

T0H2 T4H2 T12H2);

T0L1 = (i1low)+(s1low)*(0);

T4L1 = (i1low)+(s1low)*(4)+rlow*1;

T12L1 = (i1low)+(s1low)*(12)+rlow*1.4;

T0L2 = (i2low)+(s2low)*(0);

T4L2 = (i2low)+(s2low)*(4);

T12L2 = (i2low)+(s2low)*(12);

T0M1 = (i1mid)+(s1mid)*(0);

T4M1 = (i1mid)+(s1mid)*(4)+rmid*1;

T12M1 = (i1mid)+(s1mid)*(12)+rmid*1.4;

T0M2 = (i2mid)+(s2mid)*(0);

T4M2 = (i2mid)+(s2mid)*(4);

T12M2 = (i2mid)+(s2mid)*(12);

T0H1 = (i1hi)+(s1hi)*(0);

T4H1 = (i1hi)+(s1hi)*(4)+rhi*1;

T12H1 = (i1hi)+(s1hi)*(12)+rhi*1.4;

T0H2 = (i2hi)+(s2hi)*(0);

T4H2 = (i2hi)+(s2hi)*(4);

T12H2 = (i2hi)+(s2hi)*(12);

output: CINTERVAL;
